# Relationship between maternal obesity and infant feeding-interactions

**DOI:** 10.1186/1475-2891-4-17

**Published:** 2005-05-12

**Authors:** Russell Rising, Fima Lifshitz

**Affiliations:** 1 EMTAC Inc., 514 Santander Ave, #5, Coral Gables, FL 33134 USA; 2 Sansum Diabetes Research Institute, 2219 Bath St., Santa Barbara, CA 93105 USA

## Abstract

**Background:**

There are no data regarding the relationship between maternal adiposity and interaction and feeding of infants and possible contribution to childhood obesity. In this study we determined the relationship between maternal body weight and composition and infant feeding patterns and maternal-infant interaction during 24-hour metabolic rate measurements in the Enhanced Metabolic Testing Activity Chamber (EMTAC).

**Methods:**

The amount of time four obese (BMI = 33.5 ± 5.3 kg/m^2^) and three normal weight (BMI = 23.1 ± 0.6 kg/m^2^) biological mothers, spent feeding and interacting with their infants, along with what they ingested, was recorded during 24-hour metabolic rate measurements in the EMTAC. The seven infants were 4.9 ± 0.7 months, 69 ± 3 cm, 7.5 ± 0.8 kg, 26 ± 3 % fat and 29 ± 25 percentile for weight for length. Energy and macronutrient intake (kcal/kg) were assessed. Maternal body composition was determined by air displacement plethysmorgraphy and that of the infants by skin-fold thicknesses. Pearson correlations and independent t-tests were utilized for statistical analysis (p < 0.05).

**Results:**

Infants born to obese biological mothers consumed more energy (87.6 ± 18.9 vs. 68.1 ± 17.3) and energy as carbohydrate (25 ± 6 vs.16 ± 3; p < 0.05) than their normal weight counterparts. Most of the increased intake was due to complementary feedings. Twenty-four hour infant energy intake increased with both greater maternal body weight (r = 0.73;p < 0.06) and percent body fat. Furthermore, obese biological mothers spent less total time interacting (570 ± 13 vs. 381 ± 30 minutes) and feeding (298 ± 32 vs.176 ± 22 minutes) (p < 0.05) their infants than their normal weight counterparts. Twenty-four hour interaction time negatively correlated with both maternal body weight (r = -0.98; p < 0.01) and percent body fat (r = -0.92; p < 0.01). Moreover, infants of obese mothers slept more (783 ± 38 vs. 682 ± 32 minutes; p < 0.05) than their normal weight counterparts. However, there were no differences in total 24-hour energy expenditure, resting and sleeping metabolic rates (kcal/kg) for infants born to obese and normal weight biological mothers.

**Conclusion:**

Greater maternal body weight and percent body fat were associated with greater infant energy intakes. These infants were fed less frequently and consumed more carbohydrates in a shorter period of time as compared to infants from normal weight biological mothers. These variations in feeding patterns may predispose certain infants to obesity.

## Introduction

Childhood obesity is now recognized as a national health epidemic, doubling among children 6–11 years old between the last (1976–1980) and the present (1999–2000) National Health and Nutrition Examination Surveys. Obesity in early childhood leads to more severe adult obesity [1]. Overweight and obese children are facing adult onset disease type risk factors such as cardiovascular heart disease, elevated blood pressure and type II diabetes [2]. Presently, type II diabetes represents 8–45% of all new cases of diabetes diagnosed in children and adolescents [2]. This epidemic increase in childhood obesity can only lead to a lower quality and duration of life and increased health care costs [3,4].

Many studies have identified the influence of genetics and environmental factors on potential causes of childhood obesity [5,6] and [7]. Some of these include having obese biological parents [8] and biological mothers being of a low social economic status [9,10]. There are other factors in early infancy that may also contribute to obesity in childhood. For example, being born small or large for gestational age [11], breast feeding less than three months [12], exposed to early introduction of complementary foods [13] and/or excess fruit juice consumption [14]. There may be many other potential factors that may contribute to childhood obesity but as yet undiscovered.

The relationship between maternal body composition and infant and maternal interaction and feedings have not been studied. Obese biological mothers may interact in such a way as to possibly contribute to greater energy intake in their infants earlier in life. Recently we published the results of 24-hour metabolic measurements in four-to-six month old infants in the Enhanced Metabolic Testing Activity Chamber (EMTAC) [15]. This study allowed collection of data to assess the relationship between maternal body composition and infant feeding and interaction patterns.

## Methods

### Subjects

The data for this analysis were derived from a previous study of 24-hour metabolic rate and physical activity in infants. Data from seven infants were complete in regards to metabolic rate, physical activity and body composition in both the infants and biological mothers. Moreover, complete data in regards to complementary food or formula intake were available. The physical characteristics of the biological mothers are shown in Table 1 and that of their infants in Table 2. Biological mothers were classified as obese (BMI >30 kg/m^2^) or of normal weight (BMI < 24.9 kg/m^2^) [16]. The mothers and their infants were recruited from the Outpatient Clinic of Miami Children's Hospital in Miami. A complete explanation regarding the purpose, procedure, risks and benefits of the study and informed consent was obtained from the biological mother of each infant at the time of the metabolic study. The study was approved by the Institute Review Board of Miami Children's Hospital.

**Table 1:** Physical characteristics of normal and obese biological mothers

Parameter for biological mothers	Normal weight	Obese
Number of subjects	3	4
Body weight (kg)	57.8 ± 2.0	85.6 ± 12.0*
Height (cm)	158 ± 3.8	152 ± 5.1
Age (y)	27.7 ± 10.6	28.8 ± 3.2
BMI (weight;kg/height^2;^m)	23.1 ± 0.6	33.5 ± 5.3*
Body fat (%)	35 ± 2	44 ± 5*

* p <0.05

**Table 2:** Physical characteristics of infants born to normal weight and obese biological mothers

Parameter for infants	Biological Mothers	
	Normal weight	Obese

Body weight (kg)	7.4 ± 0.6	7.4 ± 1.1
Length (cm)	69 ± 6	69 ± 0.9
Age (months)	4.7 ± 0.1	5.1 ± 1.0
BMI (weight;kg/height^2;^m)	15.6 ± 1.4	15.8 ± 1.9
Body fat (%)	26 ± 2	26 ± 3
Weight for length (percentile)	27 ± 23	31 ± 30
Weight for length Z-score	-0.1 ± 0.9	0.07 ± 1.20
Weight for age (percentile)	72 ± 20	73 ± 17
Weight for age Z-score	-0.02 ± 1.20	0.02 ± 1.00
Length for age (percentile)	73 ± 23	89 ± 9
Length for age Z-score	-0.4 ± 0.6	0.39 ± 0.28

### Maternal Anthropometry

Maternal height was measured using an Ayrton Model S100 hospital grade stadiometer (QuickMedical Inc., Snoqualmie WA) and weight was determined on a digital balance (LMI Inc., Concord, CA). Thereafter, body composition was measured by air displacement plethysmography using the BodPod Body Composition System (LMI Inc., Concord CA). The principle of the method is similar to hydrostatic weighting except that body volume is obtained by air displacement instead of water. The subjects sat for two 50 second testing sessions in a 450-liter chamber. A moving diaphragm determined the difference in air pressure between where the subject sat in the front chamber and a rear reference chamber. The pressure difference, along with the subject's body weight, was used to calculate body volume. From these results body fat was calculated using the Siri equation [17].

### Infant Anthropometry

On the day of the study supine length (crown to heel) was measured in duplicate with a horizontal stadiometer (Perspective Enterprises, Kalamazoo, MI) and body weight was the average of two measurements obtained with an infant scale (Cardinal Detecto, Webb City, MO). Skin-fold thicknesses were the mean of two measures at each of five sites (biceps, triceps, sub scapular, flank and quadriceps) on the right side of the body using a Lange skin-fold caliper (Beta Technology, Cambridge, MD) according to a standard procedure [18]. Body fat and fat-free mass were calculated by appropriate equations [19].

### Maternal interaction and infant feeding patterns

Energy and macronutrient intakes were determined from the formula and infant food manufacturer's proximate analysis for the nutrient components [20]. The amount of each food consumed by the infant during the 24-hour period was recorded. The actual amount of formula consumed by the infant was determined using calibrated infant feeding bottles and the amount of complementary feeding was also determined. The recording of energy and macronutrient intake started one-hour prior to and ended one-hour before the conclusion of the 24-hour metabolic testing period. The one-hour off-set was necessary to include or exclude energy consumed before and after the metabolic testing period which compensates for variations in feedings and the intestinal transient time in this age group of infants [15].

Only the biological mothers were present and cared for, fed and interacted with infants during the entire 24-hour testing period. During metabolic testing, biological mothers continued to feed their infants at their discretion in the same manner as before the study. They brought the milk formula and complementary foods they selected to the laboratory. The biological mothers were advised not to alter their infant feeding practices for the duration of the study. All infants born to normal weight biological mothers and one from an obese biological mother were only formula fed prior to and during metabolic measurements. Three out of the four infants born to obese biological mothers were receiving complementary foods beginning just after four months of age and were fed such during the study. The complementary foods included rice cereal, mixed vegetables, Beachnut^® ^apple sauce and dessert fruit pudding. Six of the infants were fed Carnation Good Start^® ^with iron while one was fed Carnation Alsoy^®^.

One member of the research team in charge of the 24-hour metabolic study was always directly involved with the testing procedure and was in close proximity to the infant and available to parents to address questions and concerns during the 24-hour period. The investigators acted as observers and recorded infant feedings, amount and type of formula fed along with consumption of complementary foods. Upon completion of the 24-hour metabolic measurement, macronutrient intake was determined from the amount of formula or complementary food fed utilizing the manufacturer's proximate analysis. This methodology of determining nutrient intake is valid and has been utilized in several previous studies in infants [21,22] and adults [20,23]. Investigators also recorded the interaction time and type of interaction and the observed periods of infant sleep. Furthermore, any other contact with the infant during the entire 24-hour testing period was also recorded.

The mean amount of time infants spent feeding was calculated by taking the sum of the time of all feeding periods and dividing by the number of feedings over the 24-hour testing period. The mean time between feedings during the day was calculated by taking the sum of the time intervals between each feeding session from 9:30 AM -11:30 PM and dividing by the number of feedings over the same period. The day and night periods were chosen to conform to the standards of previous 24-hour metabolic studies [24,25]. The mean time between feedings for the entire testing period was calculated in a similar manner but included all feedings over the course of the 24-hour metabolic measurement.

Interaction time was calculated by taking the sum of all data summary periods were the biological mother was interacting with her infant. Each data summary period was a five minute mean of continuous measurements of energy expenditure and physical activity index. The 24-hour testing period consisted of 288 five minute data summary periods. Interaction recorded included feeding, holding and cuddling the infant as well as diaper changes. In a similar manner the total amount of interaction time by biological mothers one hour prior to feeding was calculated by taking the sum of all summary periods one hour prior to each feeding episode. Finally, the total time infants spent engaged in feeding was calculated by taking the sum of all summary periods were the infant was being fed [15]. Feeding included all formula and complementary foods.

### Measurements of metabolic rate and physical activity

**Figure 1:**
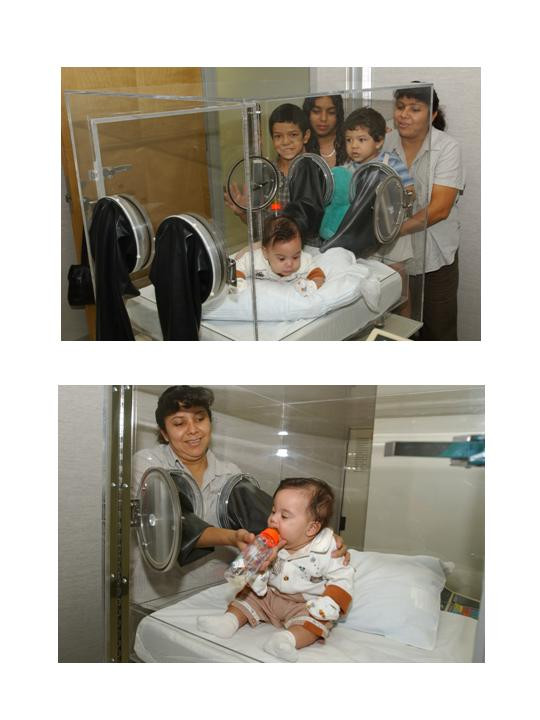
Photos showing the interaction between the biological mother and her infant utilizing the hand access ports of the EMTAC.

Biological mothers were given instruction on how to interact with their infants while in the EMTAC and was allowed time to practice using the hand access ports prior to metabolic testing (Figure 1). Once all of the instruction and instrument calibrations were completed each infant was placed in the EMTAC for 24-hours from 9:30 AM till 9:29 AM the following day for continuous measurements of energy expenditure (EE; kcal/min) and physical activity (PA; oscillations in weight/min/kg body weight) as described previously [15]. Any supplies such as diapers, formula, complementary infant foods or toys were placed in the EMTAC in hanging bags before the start of the test. The mother of the infant being studied was provided lodging within the laboratory during the entire testing period. There were no restrictions in regards to room lighting, feeding or interaction of the infant or with any of the activities of the family during the entire testing procedure.

Energy expenditure (kcal/min) was continuously calculated during metabolic testing according to the method of Jequier [25] and summarized every five minutes as described previously [26]. At the conclusion of each metabolic test, all metabolic data were corrected for parental interaction, prior to the calculation of resting (RMR; kcal/kg/day) and sleeping metabolic rates (SMR; kcal/kg/d) as described previously [15]. All metabolic results were expressed as kcal/kg body weight/day.

The small number of infants in the study was due to the difficulties in recruiting biological mothers willing to stay in or around the laboratory for almost 30 hours. This time was necessary to instruct the biological mothers on how to interact and feed their infants using the hand access ports of the EMTAC and allow practice sessions prior to the start of the 24-hour metabolic measurement. Furthermore, body size and composition measurements had to be obtained for both the infants and biological mothers prior to the metabolic test. Moreover, the EMTAC had to be prepared and calibrated prior to the start of the 24-hour metabolic measurement.

### Statistical Analysis

Person correlations were used to determine the relationship between the mother's anthropometry and interaction time and energy intake. Independent t-tests were used to determine differences in all metabolic and feeding parameters studied between obese and non-obese biological mothers. Significance (p < 0.05) was determined at the five percent level of probability.

## Results

Height and age were similar among the normal weight and obese biological mothers. The latter had a greater body weight, BMI and percent body fat (Table 1) in comparison to their normal weight counterparts (p < 0.05). Furthermore no differences existed in regards to body weight, body composition and growth performance between infants of normal and obese biological mothers (Table 2). The similarity of the infants in terms of growth performance at the time of the study was further verified by the lack of significant differences in Z-scores obtained for weight for length, weight for age and length for age percentiles. None of the growth assessment parameters of the infants were more than 0.4 standard deviations from the mean.

**Figure 2:**
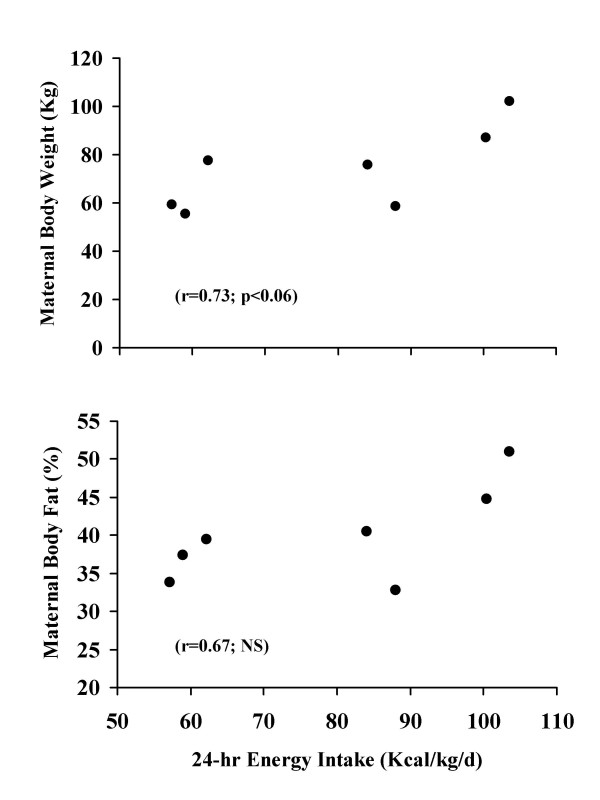
Correlation between 24-hour energy intake (kcal/kg/d) with both maternal body weight (kg; Figure 1a top plot) and body fat (%; Figure 2b bottom plot) for the seven infants in this study.

Infants born to obese biological mothers consumed more energy, and energy as carbohydrate, than their normal weight counterparts (Table 3). Three, out of the four infants born to obese biological mothers consumed complementary foods. The amount of energy consumed from complementary foods by these infants of obese biological mothers was 18.3 ± 2.5 kcal/kg. This was in addition to the energy intake of 69.1 ± 20.3 kcal/kg from formula for these same infants. However, energy intake from protein and fat, for both complementary feedings and formula, were similar among the two groups (Table 3). The amount of formula intake was also similar (90.1 ± 16.3 vs. 98.9 ± 35.4 ml/kg) between the infants born to obese and the normal weight biological mothers. There was a significant (p < 0.05) correlation between total energy intake and maternal body weight (r = -0.73; p < 0.06; Figure 2a).

**Table 3:** Nutrient intake and feeding profile for the infants born to normal weight and obese biological mothers

Parameters for infants	Biological Mothers	
	Normal weight	Obese

Energy intake from formula (kcal/kg)	68.1 ± 17.2	69.1 ± 20.3
Energy intake from complementary foods (kcal/kg)	0	18.3 ± 2.5
Total 24-h energy intake (kcal/kg body weight)^1^	68.1 ± 17.2	87.6 ± 18.9
Formula intake (ml/kg body weight)	90.1 ± 16.3	98.9 ± 35.3
Energy intake from CHO (kcal/kg body weight)^2^	16 ± 3	25 ± 6*
Energy intake from PRO (kcal/kg body weight)^3^	4 ± 1	6 ± 3
Energy intake from FAT (kcal/kg body weight)^4^	38 ± 10	38 ± 12
Energy intake per feed (kcal/kg body weight)	7.4 ± 1.2	12.9 ± 4.8
Number of feedings (#)	9.3 ± 2.1	7.3 ± 2.2
Duration of each feeding (min)	33 ± 6	26 ± 5
Average time between feedings over 24-hours (min)	127 ± 28	188 ± 39
Average time between feedings over the day (min)	88 ± 32	108 ± 43
Total 24-hr total feeding time (min)	298 ± 32	176 ± 22*
Total 24-hr interaction time (min)	570 ± 13	381 ± 58*
Total interaction time one hour prior to feedings (min)	157 ± 29	68 ± 34*
Number of infants who had night time feedings	3	3
Total sleeping time (min)	682 ± 32	783 ± 38*

* = p < 0.05

^1 ^24-hr EI = Twenty four hour energy intake expressed per kg body weight

^2 ^CHO = Carbohydrate expressed per kg body weight

^3 ^PRO = Protein expressed per kg body weight

^4 ^FAT = Fat expressed per kg body weight

**Figure 3:**
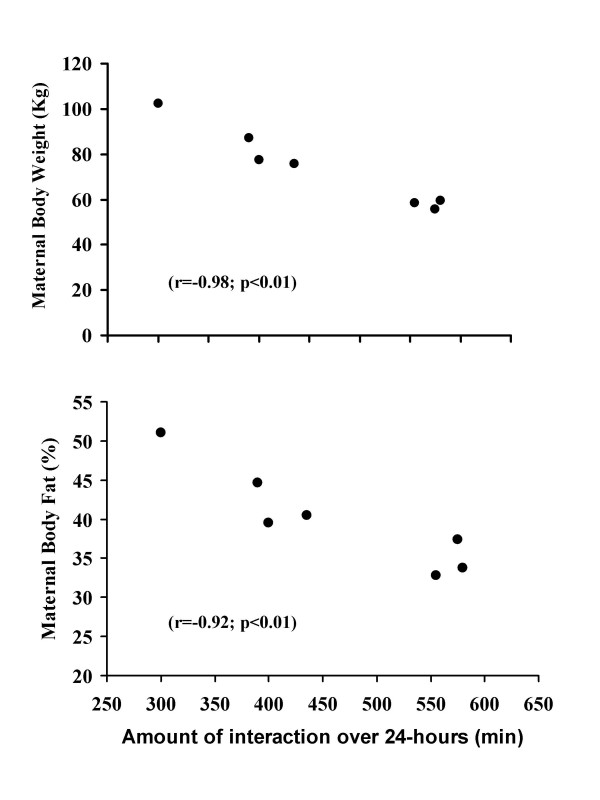
Correlation between interaction time (minutes) with both maternal body weight (kg; Figure 1a top plot) and body fat (%; Figure 1b bottom plot) for the seven infants in this study.

**Figure 4:**
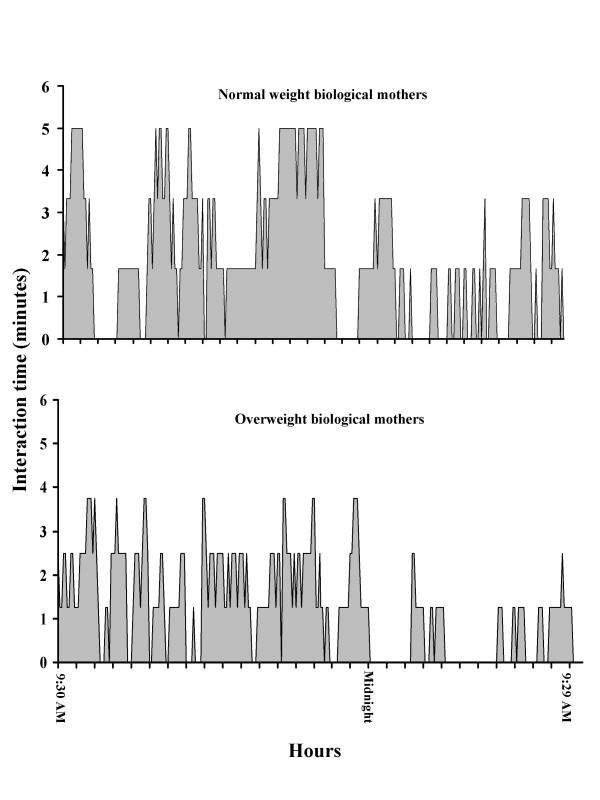
Twenty-four hour interaction profile for infants born to normal (top) and obese (bottom) biological mothers. The Y-axis represents the mean amount of interaction time over each five minute period. There are 288 five minute summary periods during a 24-hour metabolic measurement. A Obese biological mothers spent less time (p < 0.05) interacting with their infants during the day (9:30 AM till 11:30 PM) than their normal weight counterparts. Similar differences occurred during the evening hours (11:31 PM till 5:30 AM) but the differences were not significant.

Obese biological mothers spent less time interacting and feeding their infants over the course of the 24-hour testing period (Table 3). There was a negative correlation between total 24-hour interaction time and both maternal body weight (r = 0.98; p < 0.01, Figure 3a) and body fat (r = 0.92; p < 0.01, Figure 3b). Moreover, overweight biological mothers spent less time interacting with their infants one hour prior to feeding (Table 3). The pattern of maternal interaction over the course of the 24-h testing period is shown in Figure 4. Normal weight biological mothers interacted with their infants more over the course of the 24-hour testing period than the obese biological mothers (Figure 4). When considering just the hour prior to each feeding, normal weight biological mothers interacted with their infants more than their obese counterparts (Table 3). The increased interaction was more significant (p < 0.05) during the day time hours (9:30 AM till 11:30 PM) while during the evening hours (11:31 PM till 5:30 AM) the difference in interaction time between normal and obese biological mothers was less significant (p < 0.10; Figure 4). Finally, infants of overweight biological mothers spent more time sleeping (Table 3) than their normal weight counterparts.

The number of feedings and the amount of time necessary for each meal tended to be less for the infants of obese biological mothers, however, these differences were not significant (Table 3). There was also a tendency for infants of obese biological mothers to consume more energy at each feeding in comparison to those of normal weight mothers. Furthermore, infants of obese biological mothers tended to allow more time in between feedings during the day and over the entire 24-hour testing period (Table 3).

There were no significant differences in any of the metabolic parameters between infants from obese and normal weight biological mothers (Table 4). However, there was a tendency for a higher respiratory quotient and a greater amount of physical activity in those infants born to obese biological mothers.

**Table 4:** Twenty-four hour metabolic rate and physical activity of infants born to normal weight and obese biological mothers

Parameters for infants	Biological Mothers	
	Normal weight	Obese

24-hr EE (kcal/kg body weight)^1^	76.0 ± 5.2	74.3 ± 4.3
RMR (kcal/kg body weight)^2^	65.0 ± 5.0	64.9 ± 3.4
SMR (kcal/kg body weight)^3^	63.0 ± 4.3	64.6 ± 4.1
24-hr PA (oscillations/min/kg body weight)^4^	2.9 ± 0.4	5.0 ± 2.4
RQ (VCO_2_/VO_2_)^5^	0.84 ± 0.03	0.89 ± 0.02

^1 ^24-hr EE = Twenty four hour energy expenditure expressed per kg body weight

^2 ^RMR = 24-hour resting metabolic rate expressed per kg body weight

^3 ^SMR = 24-hour sleeping metabolic rate expressed per kg body weight

^4 ^PA = physical activity as determined by the amount of oscillations in weight (g) derived from the balance within the EMTAC enclosure. Data includes periods of sleep

^5 ^RQ = Respiratory quotient determined as the rate of carbon dioxide production/oxygen consumption in liters

## Discussion

In this study we demonstrated through direct observation that infants born to obese biological mothers ingested more energy and a greater amount of that energy were derived from the carbohydrates present in complementary foods. Furthermore, increased maternal body weight and fatness were related to increased 24-hour energy intake. Moreover, increased body weight and fatness of the biological mothers resulted in a decline in the amount of time spent interacting with their infants during the 24-hour testing period. This included less time spent feeding the infant, and particularly a lower amount of interaction one hour prior to feedings.

The biological mothers were able to freely interact with their infants in a comfortable setting. However, none of the biological mothers reported being uncomfortable with their infant being in the EMTAC or staying at the laboratory during the metabolic testing. Furthermore, they did not report any difficulties in interacting or feeding their infant utilizing the hand access ports of the EMTAC. Nonetheless, it is possible that the unfamiliar surroundings of the metabolic laboratory might have contributed to some changes in infant feeding practices not present in their maternal home setups.

Excess energy consumption early in an infant's life of those born to obese mothers, possibly accelerated with complementary food intake, might set the stage for future childhood obesity. In this study, there were no significant differences in infant body weight or composition between four to six months of age among the two groups of infants studied. However, infants of obese biological mothers consumed an average of 19.7 kcal/kg body weight more than the infants born to normal weight mothers during this study. Assuming that approximately 4900 kilocalories are needed per kilogram of body weight gain [27], it would take the infants of obese biological mothers a considerable amount of time to become obese if they would continue ingesting this amount of excess calories each day. This might explain why investigators report that it takes up to two years before a noticeable gain of body fat is observed in young children [1,28]. In overfed adults 66% of body weight gain is fat while the remainder is fat-free mass [29]. It is possible that overfeeding infants over a long period of time causes excess body weight gain of similar composition. However, no studies to date have quantified the composition of body weight gain in overfed infants from the time of birth.

Early introduction of complementary foods might increase body weight gain. In one study, early introduction of complementary foods was associated with greater infant body weight gain [30]. Other investigators [31] reported that complementary foods introduced to infants between 9 and 16 weeks showed a slight increase in weight gain velocity (g/week) in comparison to those infants who were introduced to these foods after 25 weeks. Both of these studies [30,31] suggest that early introduction of complementary foods might increase body weight gain. Therefore, early introduction of complementary foods, coupled with the increased energy intake at each feeding in the infants from obese biological mothers, as seen in our study, might set the stage for future childhood obesity. However, we did not have data to ascertain why obese mothers started complementary feedings in their infants.

Infants may be introduced to complementary foods at different times. In the Feeding Infants and Toddlers Study (FITS), 71% of the parents reported introducing complementary foods to their infants between four and six months of age while the other 29% reported introducing these foods to their infants at less than four months [32]. In another study were the National Health and Nutrition Examination Survey (NHANES III) data were analyzed, less than 25% of the parents reported feeding complementary foods to their infants prior to four months of age [33]. Moreover, the parents in both of these studies [32,33] were similar in regards to social economic status, age and ethnic background. In our study we did not have data on why obese biological mothers were feeding complementary foods to their infants. Moreover, neither the FITS [32] nor the NHANES III [33] studies related infant feeding practices to maternal body composition.

There were no significant differences in any of the metabolic parameters measured such as 24-hour energy expenditure, resting and sleeping metabolic rates, respiratory quotient and the index of physical activity. Moreover, there were no differences in any of the growth parameters (weight for length, weight for age and length for age percentiles) between the two groups of infants at the time of the study. Our results are in agreement with Stunkard et al [28] who found no differences in growth parameters, body composition, total energy expenditure by doubly labeled water, sleeping metabolic rate or physical activity in infants born to obese (greater than the 66^th ^percentile for BMI) or lean (<33^rd ^percentile for BMI) biological parents throughout the first year of life. In contrast to our results and those of Stunkard [28], Roberts et al [8] found reduced total daily energy expenditure in infants born to obese parents as determined by the doubly-labeled water method. The reduction of total daily energy expenditure was due to less physical activity in these infants [8]. It is possible that less interaction between obese mothers and their infants [8] accounted for the reduction in physical activity.

Since infants gain approximately 5 g/kg of body weight per day at four months of age [34], it is possible that constant additional caloric intake of those infants born to obese biological mothers will be manifested as additional daily body weight gain later in life. All these data suggest that maternal influences on infant body composition may not appear initially as obvious physical differences during the first six months of life. It is possible that the differences detected among biological obese mothers and their infants could affect the body composition of their infant as they age.

There may be other factors beginning in infancy that may be associated with the eventual increase in adiposity in later life. For example, fewer, but larger feeds and a higher sucking pressure were associated with greater adiposity in toddlers at two years of age [13]. This is in partial agreement with our results were we found that obese biological mothers spent less time interacting and feeding their infants. Moreover, infants from obese biological mothers consumed more energy in less time at each feeding. It is possible that the infants were hungrier due to the longer time between feedings.

Another study reported that greater maternal BMI during the first trimester of pregnancy was related to a higher prevalence of obesity in children from two to four years old. This is equivalent to 1 out of 4 children of obese mothers becoming obese as opposed to only 1 out of 10 children from normal weight mothers [35]. Moreover, it was also reported that a greater maternal BMI was a modest predictor of their daughter's relative weight at five years of age [36]. All of these studies [13,28,36] relate possible maternal influences upon future obesity of their infants. However, none eluted to the actual difference in the care of infants from either normal or obese mothers such as found in our analysis.

The association between the physical characteristics of biological mothers and their infants has not been ascertained. Six studies found no relationship between maternal BMI and infant's body weight after the first year of life [35,37-41] while two found such a relationship [42,43]. None of these studies accurately measured maternal body composition and did not include direct observation of the interaction dynamics between mothers and their infants. This was done in our study using air displacement plethysmography for maternal body composition and direct observation of food intake and feeding patterns including the number and length of infant feedings and the amount consumed at each meal during the entire 24-hour period. Additionally the type and length of maternal interaction with their infants revealed significant differences between normal weight and obese biological mothers which may not have been elucidated by other means. Though there were a small number of infants studied the results suggest that differences do exist on how mothers interact with their infants, depending on their body composition.

## Conclusion

This study provided some new insight as to possible influences, beginning in infancy, as to the possible causes of childhood obesity. We have found additional factors that may contribute to future childhood obesity. The ability to conduct 24-hour metabolic rate measurements and direct accurate recordings of the biological mother's interaction with their infants elucidated specific differences in the care of infants that were related to maternal body weight and adiposity.

## Abbreviations

EMTAC = Enhanced metabolic testing activity chamber

ANOVA = Analysis of Variance

BMI = Body mass index

EE = Energy expenditure

EI = Energy intake

RMR = Resting metabolic rate

SMR = Sleeping metabolic rate

PA = Physical activity index

RQ = Respiratory quotient

CHO = Carbohydrates

PRO = Protein

FITS = Feeding Infants and Toddlers Study

NHANES = Nutrition Health and Nutrition Examination Survey

## Competing interests

The author(s) declare that they have no competing interests.

## Authors' contributions

Dr. Russell Rising has contributed to the design of the experiment and conducted the data analysis. Furthermore, he either participated in some of the actual data acquisition or supervised pediatric research fellows in this regard. He also assisted in the preparation of the small grants necessary for funding of this project. Finally, he also assisted in the writing and editing of this manuscript.

Dr. Fima Lifshitz directed the research and contributed to the preparation of the manuscript and assisted with data analysis. He also generated some of the grant proposals necessary for the financial support of this study. Both authors were involved in the final writing of this manuscript.
